# Comparison of Wide Conjunctival Flap and Conjunctival Autografting Techniques in Pterygium Surgery

**DOI:** 10.1155/2013/209401

**Published:** 2013-03-03

**Authors:** Lokman Aslan, Murat Aslankurt, Adnan Aksoy, Murat Özdemir, Erdem Yüksel

**Affiliations:** Ophthalmology Department, Faculty of Medicine, Sutcu Imam University, 46100 Kahramanmaraş, Turkey

## Abstract

Pterygium is an abnormal fibrovascular tissue extending on the cornea which is a degenerative and hyperplastic disorder. A stromal overgrowth of fibroblast and blood vessels is accompanied by an inflammatory cell infiltrate and abnormal extracellular matrix accumulation. The surgical excision is the main treatment method of pterygium, but recurrence is the most common postoperative complication. In the present study, we aimed to compare the wide conjunctival flap and the conjunctival autografting techniques in pterygium surgery according to time of operation, safety, and effectiveness. Results showed that the effect of wide conjunctival flap techniques on primary pterygium surgery was found close to the conjunctival autograft techniques. In addition, the flap technique has a shorter surgical time, the surgery does not require extreme experience, feeding of the flap is provided with own vessels since the vascular structure is protected on the upper temporal conjunctival area, reverse placement of the flap is not seen, it needs fewer sutures, so that suture disturbances may reduce, and it is less traumatic than autograft technique during conjunctival transport. Therefore, this technique may be preferred in suitable cases.

## 1. **Introduction**

Pterygium is an abnormal fibrovascular tissue extending on the cornea which is a degenerative and hyper plastic disorder [1]. A stromal overgrowth of fibroblast and blood vessels is accompanied by an inflammatory cell infiltrate and abnormal extracellular matrix accumulation [2]. Recently authors have focused on ultraviolet rays whether it could be any changes in limbal stem cell [3]. Although several hypotheses have been associated with etiology, its pathology still remains to be explained [4]. Some individuals or occupational groups are susceptible to this disease. It is more common in drivers, welders, and carpenters, the countries with relatively high exposure to ultraviolet radiation, the hot and dusty climates, and those living in rural areas [3, 4]. While the body of the pterygium remains on the sclera, the head advances onto the cornea in many cases affecting vision, causing general discomfort, and becoming a cosmetic nuisance [5]. 

The surgical excision is the main treatment method of pterygium. The various surgical techniques such as bare sclera, conjunctival autografting, primary conjunctival closure, intraoperative mitomycin C application, and amniotic membrane transplantation have been applied [6, 7]. Recurrence is the most common postoperative complication [8]. Although many techniques have been tried in order to prevent it, perfect technique has not been found until now [7]. Other uncommon and more easily managed complications of pterygium surgery are corneal epithelial defects, dellen formation, suture loosening, graft edema, and pyogenic granuloma [8]. The rare complications of symblepharon and diplopia may be associated with the excision of multi recurrent pterygia [8]. We compared conjunctival autograft technique and wide conjunctival flap technique in terms of their efficacy, safety, and operation time in the present study. 

## 2. **Material and Methods**

The study was approved by local ethics committee and conducted in accordance with the ethical principles described by Declaration of Helsinki. Informed consent was obtained from participants.

Full ophthalmological examination including the best corrected visual acuity, slit lamp examination, intraocular pressure measurement, and funduscopy was performed in all subjects. Patients with primary pterygium were enrolled to study and created two groups. The patient was randomly selected through consecutive surgery.

Operation was made by the same surgeon (LA) under local anesthesia. The operating microscope was used. After instillation of topical anesthetic drops on patient's eye, lids were ruled out with blepharostat or lid speculum. Lidocaine hydrochloride 20 mg/mL + epinephrine 0.0125 mg/mL (2% Jetokain amp, Adeka, Turkey) approximately 1 mL containing local anesthetic was injected with 25-gauge needle into the suprascleral space beneath pterygium. Penetration between the sclera and pterygium of corneoscleral limbus was separated with an iris repository. Cornea was engraved by blunt knife. The pterygium head and body were dissected from both the surface of the sclera and overlaying conjunctiva and then excised. Sclera was cauterized, but excessive cauterization was avoided. 

**Figure 1:**
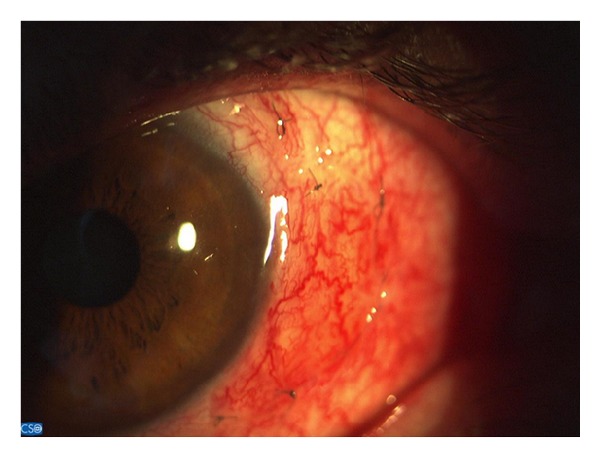
The conjunctival autograft technique was performed in the first week after surgery.

In the first group, the dimensions of bare sclera were measured. Superior temporal bulbar conjunctiva estimated was approximately 1-2 mm greater than bare sclera size. Conjunctival graft was dissected from the tenon for obtaining the thinnest possible conjunctiva. Limbal portion of the graft was sutured with 10/0 nylon suture to the side of the cornea. Both ends of the graft were adjusted with single suture at the inferior and superior limbus and the other side, with continuous suture circular manner. The autografting area was closed from patient's own bulbar conjunctiva (Figure 1).

**Figure 2:**
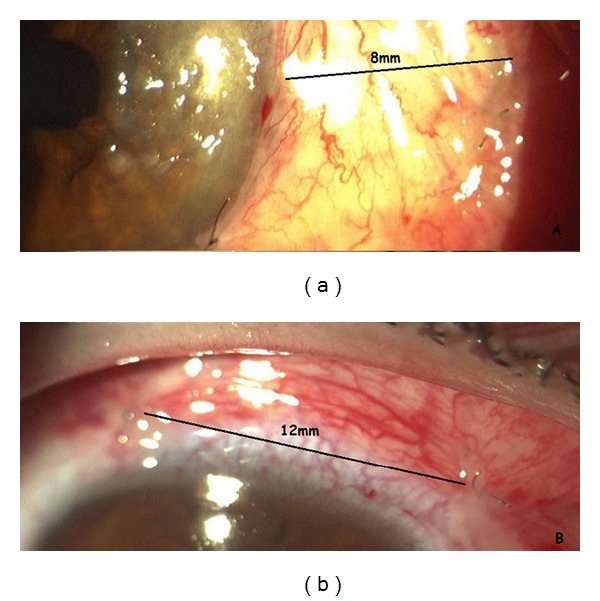
The flap size of conjunctival flap technique; (a) the width of flap is 8 mm and (b) the length of flap is 10–12 mm.

**Figure 3:**
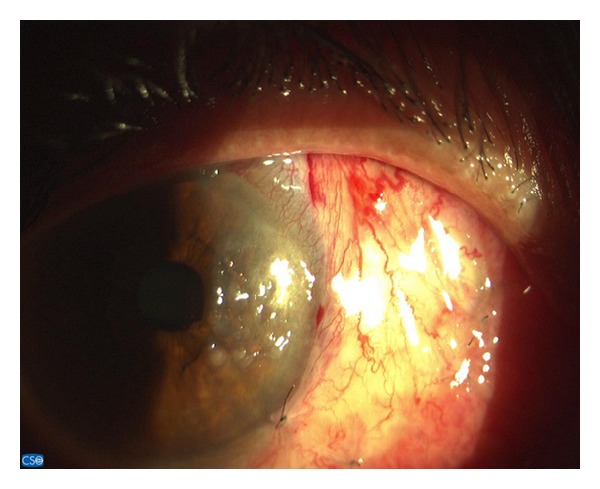
The wide conjunctival flap technique was performed in the first week after surgery.

In the second group, wide conjunctival flap was removed from the superior bulbar conjunctiva near the limbus. We wanted to obtain a wide, rectangular shape, 7-8 mm in width and 10–12 mm in length flap (Figures 2(a) and 2(b)). The flap was then thinly dissected from Tenon's capsule and was transposed to the pterygium excision site. The flap was placed at least 1 mm of bare sclera adjacent to the cornea-scleral limbus, and then two separate sutures were performed at inferior and nasal site (Figure 3). The cut edges of the conjunctiva on the donor site were closed with two separate sutures (Figure 2(b)). Fibrosis was not observed on flap taken area at follow-up period.

Antibiotic ointment was applied; then the eye was closed with an eye roundel. Postoperative antibiotic and corticosteroid drops were given for about 2 weeks. All sutures were removed one week after the surgery.

## 3. **Results**

The first group consisted of 22 patients (12 males and 10 females), mean age 45.25 ± 17.14 years, and the second group was 23 patients (12 males and 10 females), mean age 48.21 ± 15.13 years. Demographic characteristics were shown in Table 1. There were not statistically significant differences in age and gender distributions of groups (*P* = 0.523 and *P* = 0.752, resp.). 

The average operation time was 25.7 ± 2.31 minutes in the first group and 16.4 ± 1.84 minutes in the second group. The first group had a longer surgical time than the second one and this difference was statistically significant (*P* < 0.001).

Postoperative recurrence was seen in two cases (9%) in the first group, at 12.9 ± 3.2 months of follow-up period, and it was three cases (13%) in the second group at 13.2 ± 3.8 months of follow-up period. Dellen was observed in one case (4.5%) of the second group within the first week, it was not seen in the first group during follow-up. Suture loosening was observed in four cases (18%) in the first group, and it was in one case in the second group (4.3%). Graft edema was in two (9%) cases in the first group, but flap edema was not seen in the second group. There was not any conjunctival or scleral necrosis in both groups (Table 2). The complication rate of surgery compared between groups was not statistically significantly different (chi-square test, *P* = 0.465).

## 4. **Discussion**

A conservative treatment is sufficient to resolve complaints arising from an early stage pterygium, but such therapy is symptomatic and temporary [1, 9]. The artificial tears or immunomodulatory agents such as a cyclosporine drop can be used for this purpose and provide comfort and relief from foreign body sensation [9, 10]. Also, short-term anti-inflammatory eye drops may also be useful for inflamed pterygia [9]. Additionally bevacizumab, which is antineoplastic agent has been used for the treatment of pterygium recently [11, 12]. Enkvetchakul et al. have used intralesional bevacizumab injection on primary pterygium to alleviate the symptoms. They reported that reduction of pterygium tissue and vascular regression were seen [11]. However, all medical treatments have not eliminated the indication of surgery [9, 13, 14]. If pterygium progresses and leads to visual impairment either because of induced astigmatism or encroachment onto the visual axis, marked cosmetic deformity, marked discomfort and irritation unrelieved by medical management, and limitation of ocular motility secondary to restriction, surgical treatment then becomes a requirement [1, 9]. The complete excision of a pterygium from the cornea and sclera, subsequently leaving a bare denuded corneoscleral surface, is the classical surgical procedure. This procedure, also known as the bare sclera technique, was first fully described by D'Ombrain in 1948 [15]. This technique is much easier than others in practice and does not require more surgical experience [7]. It has been the treatment choice for pterygia for a long time, but this procedure has led to search for adjunctive treatment options due to the high frequency of recurrence [16]. Moreover this technique breaks down the integrity of the ocular surface and causes more complications, so it is less preferred now [16].

Postoperative recurrence is the most prominent undesirable nuisance in the pterygium surgery. The risk factors of pterygium recurrence are not distinctively known, but a number of factors such as pterygium type, age of patient, environment, reexcision of a recurrent pterygium, and surgical technique may be responsible [17]. The recurrence rate varies according to the technique used, and it has been reported in different rates. Bare sclera technique was 45–90%, conjunctival autograft technique was 3%–28%, and sliding conjunctival flap technique was 3–33% [9, 17]. Recurrence rate of the present study was 9% in conjunctival autograft and 13% in wide conjunctival flap. The recurrence of the pterygium usually occurs during postoperative first six months. Other postoperative complications such as corneal epithelial defects, dellen formation, suture loosening, graft edema, necrosis, and pyogenic granuloma may mostly be removed with simple medical or surgical treatments. Nonrecurrence complications rate on pterygium surgery was investigated by Onay et al. [18], and they reported that these complications increased in preoperative antimetabolite application and over cauterization. 

 Some adjuvants have been used preoperatively or postoperatively in order to prevent recurrence. Topical cyclosporine 0.05% has been shown to be effective in a postoperative application [19]. Mitomycin C, an antineoplastic agent, is used as an adjuvant to surgery either as intraoperative application or postoperative eye drops [20, 21]. However, Rubinfeld et al. [22] revealed that adjunctive mitomycin C therapy may cause many complications such as scleral ulceration, necrotizing scleritis, perforation, iridocyclitis, cataract, infection, glaucoma, scleral calcification, and loss of an eye. Mitomycin C has been limited to use in pterygium surgery because of such adverse effects [22]. Hence adjuvant agents were not used in the present study. 

Yu et al. [23] reported that they studied three techniques including amniotic membrane transplantation, corneal stem-cell autograft, and pedicle flap transposition and did not find any significant difference between them. Although amniotic membrane transplantation in pterygium surgery has been found effective by many researchers [23, 24], its obtainability is difficult, and application requires experience and has risk for disease transmission. 

Conjunctival autograft has gradually become a popular treatment for pterygium since 1985. In this technique, the bare sclera is covered by using autolog conjunctival tissue [6, 17]. The conjunctival autograft technique is the most effective in reducing recurrence more safely, but surgical time is longer, and various nonrecurrence complications have been demonstrated such as postoperative suture discomfort, graft edema, and graft separation [25]. Some researchers have used suture instead of tissue glue in order to reduce the negative effects of this technique. Koranyi et al. and Pan et al. have used fibrin glue in a study group and absorbable sutures in controls [25, 26]. They observed significantly lower surgery time, fewer postoperative complaints, and less recurrence rates in the fibrin glue group. However, the fibrin glue is costly, difficult to obtain, and may cause allergic reactions [26]. 

Excised pterygial area has been closed by many surgeons with different flap methods until now [17, 23]. One of the applied methods is simply sutured in free ends of the conjunctiva. Although this technique is an easier, applicable method, there are more postoperative complaints and complications in this technique [9]. The vertical conjunctival bridge flap, that stretched a rotation flap from upper bulbar conjunctiva to inferonasal edge, was applied by Kaya and Tunç [27]. They have reported that the mentioned technique has positive results, compared to bare sclera technique. In the present study, we used a wider conjunctival flap than current flap technique. Unlike an ordinary rotation flap, the wider flap was taken from upper bulbar conjunctiva, and conjunctival flap was adjusted with the lower nasal edge. By using this technique we closed a large scleral area regularly and provided a tightly conjunctival surface. The surgical time of wide conjunctival flap technique was found less than autograft technique. The shortening surgical time contributes to patient comfort, and it means less surgical manipulation, so trauma-induced complications such as graft edema and hemorrhage were seen less commonly. Unlike autograft, feeding of the flap is provided with the own vessels in the flap technique since the vascular structure is protected on the upper temporal conjunctival area. Additionally, reverse placement of the flap is not seen in this technique. In addition, this technique needs fewer sutures, so that suture disturbances may be reduced, and it is less traumatic than autograft technique during conjunctival transport. The disadvantage of this technique, limbal side of rotational flap, remains away from the cornea, and an extensive conjunctival tissue is taken. 

As a result, the effect of wide conjunctival flap techniques in primary pterygium surgery was found close to the conjunctival autograft techniques. The short surgical time and the surgery do not require extreme experience. This technique may be preferred in suitable cases.

## Figures and Tables

**Table 1:** Age, sex, and operation time values in the study groups.

Groups	Age	Gender		Operation time
	Mean ± SD (range)	M	F	Mean ± SD (range)
Group 1	45.25 ± 17.14 (21–75)	10	12	25.7 ± 3.31 (21–35)
Group 2	48.21 ± 15.13 (22–78)	13	10	16.4 ± 1.84 (14–21)
*P*	0.523	0.752		<0.001

M: male, F: female.

**Table 2:** Distribution of the complications in groups.

Groups	Recurrence	Dellen	Suture loosening	Graft/flap edema	Corneal/scleral necrosis	*P*
	*N* (%)	*N* (%)	*N* (%)	*N* (%)	*N* (%)	
Group 1	2 (9%)	1 (4.5%)	4 (18%)	2 (9%)	0 (0%)	0.465
Group 2	3 (13%)	0 (0%)	1 (4.3%)	0 (0%)	0 (0%)	

*N*: number of cases.
